# Impact of outpatient SARS-CoV-2 infections in minority children

**DOI:** 10.1097/MD.0000000000024895

**Published:** 2021-02-26

**Authors:** Vanessa Denny, Niva Shah, Karolina Petro, Karishma Choksey, Elizabeth DeSantis, Molly Hintz, Shruthi Rethi, Sarah Sanchez, Bernadette Sylla, Stephanie Chiu, Christina Gagliardo, Neeraja Kairam, Eberechi Nwaobasi-Iwuh, M. Cecilia Di Pentima

**Affiliations:** a Department of Pediatrics, Goryeb Children's Hospital; b Atlantic Center for Research, Atlantic Health System, Morristown, New Jersey; c Department of Pediatrics, Sidney Kimmel Medical College at Thomas Jefferson University, Philadelphia, PA; d Department of Emergency Medicine, Atlantic Health System, Morristown, New Jersey.

**Keywords:** adolescents, children, coronavirus, COVID-19, epidemiology, infectious diseases, SARS-CoV-2

## Abstract

Data regarding COVID-19 in the adult population and hospitalized children is rapidly evolving, but little is known about children infected with severe acute respiratory syndrome coronavirus 2 who do not require hospitalization.

In an observational, retrospective study we analyzed risk factors, demographics and clinical course of non-hospitalized patients ≤ 21 years of age with COVID-19 infection.

Of the 1,796 patients evaluated, 170 were infected, and 40 participated in a telephone survey. Children older >10 years of age (OR: 2.19), Hispanic ethnicity (OR: 3) and residing in counties with higher rates of poverty (OR: 1.5) were associated with higher risk of infection, while older girls were more likely to experience prolonged duration of symptoms (median: 32 days). Consistent with prior reports, fever and cough were present in most of our patients. Shortness of breath, diarrhea, anosmia, and ageusia were more common in our outpatient population than previously reported.

Larger studies addressing the clinical and psychosocial impact of CoVID-19 infection in children living in high-risk environments are warranted.

## 1 Introduction

The global severe acute respiratory syndrome coronavirus 2 (SARS-CoV-2) pandemic has widely affected adults. Of reported cases as of April 2020, only 1.7% were children <18 years old.^[1]^ By the end of August 2020, more than 476,000 pediatric cases have been reported by states across the USA, representing 9.5% of all cases.^[2]^ The impact of SARS-CoV-2 on non-hospitalized children remains limited.^[3–5]^ We sought to examined the clinical impact and indicators of population disparities among non-hospitalized children with COVID-19 in northern New Jersey.

## 2 Methods

From March 15 to June 1, 2020, patients ≤21 years old evaluated for SARS-CoV-2 infection by RT-PCR at Atlantic Health System (AHS) were identified by the laboratory department. Hospitalized patients and those tested for pre-procedure screening were excluded. Electronic records were reviewed for demographics, testing site and date. SARS-CoV-2 RT-PCR was obtained via nasopharyngeal swab and performed by BioReference, Mayo Clinic or AHS Laboratories.

At least one attempt was made via an AHS landline to contact positive patients. Verbal consent was obtained for study participation. Participants were asked standardized questions from an institutional review board (IRB) approved questionnaire in English or Spanish. Data was entered into a REDCap database. County-level rates of population living in poverty were derived from the Census Reporter.^[6]^ Descriptive statistics were used to compare demographics. Unpaired t-test was used to compare continuous variables. Odds ratios (OR) were used to assess risk among populations.

This study was approved by the AHS IRB.

## 3 Results

During the study period, 1796 outpatients ≤ 21 years old were evaluated for COVID-19 (median age: 14; interquartile range [IQR]: 5–19). Of these, 170 tested positive (9.5%), and 40 participated in the survey (24%). Patients who tested negative were younger (median age: 14; IQR: 6.75–18.25) than those infected (median age: 19; IQR: 13.25–20; *P* < .001). Gender proportions were similar in both groups (female: 47% and 44%, respectively; *P* = .5). Patients >10 years of age (OR: 2.19; 95% confident interval [CI]: 1.5–3.2) and from counties with >15% of residents <18 years of age living below the poverty line (OR: 1.5; 95% CI: 1.06–2.1) were more likely to have COVID-19. A similar ratio was associated with counties with ≥10% of their population living in poverty (OR:1.46; 95% CI:1.03–2.05). Hispanic/Latino patients were three times more likely to be infected (OR:3; 95% CI:2.29–4.03).

The median age of the 40 participants of this study was 16 years old (IQR: 8.5–19), with 52.5% being female. Fifty percent were of Hispanic/Latino descent, and 23.7% were Black. Among the 23 patients with a known contact for COVID-19, 56.5% were household members, 4 of whom were healthcare workers (30.8%). Of the four known contacts who required hospitalization, three required intensive care, and one died. Most participants had private (19; 37.5%) or U.S. government (15; 37.5%) health insurance (34; 85%). Among survey participants, 11 (27.5%) reported having an underlying health condition. The most common comorbidity was noted to be asthma (17.5%), followed by seizure disorders and insulin dependent diabetes mellitus (5%), growth-hormone deficiency, leukemia, thalassemia, and ventilatory dependency (1%). Notable risk factors among our participants were a history of smoking/vaping and obesity, present in 17.5% and 15% of our participants respectively.

Clinical manifestations and management are described in Table 1. Most patients presented with lower respiratory infection (57.5%). Duration of symptoms was available for 24 patients (median 7 days; IQR: 4–21). Girls >10 years (median 16 years) were more likely to have symptoms lasting >7 days (OR: 9; 95% CI: 1.2–63.8) with a median of 32 days (IQR: 32–47). Underlying medical conditions and government insurance were not associated with prolonged illness (OR: 0.4; 95% CI 0.03–4.6 and OR:0.89, 95% CI: 0.2–4.6, respectively). Ethnicity did not reach statistical significance regarding duration of symptoms (Hispanics OR: 2.7; CI 0.5–14).

**Table 1:** Clinical features and outpatient therapies.

Symptoms experienced during the illness	N = 40 (%)
Fever	25 (62.5)
Chills	11 (27.5)
Cough	19 (47.5)
Productive cough	3 (7.5)
Shortness of breath	17 (42.5)
Wheezing	2 (5)
Chest pain	5 (12.5)
Sore throat	11 (27.5)
Rhinorrhea	10 (25)
Abdominal pain	8 (20)
Nausea	14 (35)
Emesis	6 (15)
Diarrhea	14 (35)
Myalgias	12 (31.5)
Arthralgia	5 (12.5)
Fatigue/weakness	14 (35)
Difficulty walking or crawling	1 (2.5)
Headaches	15 (37.5)
Seizures	2 (5)
Eye pain	1 (2.5)
Syncopal event	1 (2.5)
Altered awareness/confusion	1 (2.5)
Rash	2 (5)
Loss of sense of smell	12 (30)
Loss of sense of taste	15 (37.5)
Edema of hands or feet	1 (2.5)
Treatment	
Azithromycin	2 (5)
Hydroxychloroquine	1 (2.5)

## 4 Discussion

Data surrounding COVID-19 infection has been rapidly evolving, however much of the literature is based on adults and hospitalized children.^[7]^ To the best of our knowledge, this is the first report to survey the impact of SARS-CoV-2 infections among non-hospitalized pediatric patients. In our experience, the median age of infected patients was 19 years, older than previously reported.^[1,8]^ In the early phases of the pandemic, limited testing capabilities did not allow testing of children with mild symptoms without known COVID-19 contacts or underlying risk factors. This might have resulted in underestimation of the rate of infection in non-hospitalized younger children. In our health-care system, older age, Hispanic ethnicity, and living in counties with higher rates of poverty were found to be predictors of infection.^[9]^

Although children are just as likely to contract SARS-CoV-2, about 20% of pediatric patients remain asymptomatic.^[5,10]^ Consistent with prior reports, fever and cough were present in most of our patients.^[3]^ Shortness of breath, diarrhea, anosmia, and ageusia were more common in our outpatient population than previously reported.^[3,5]^ Median duration of symptoms was similar to adults, but older girls were more likely to experience prolonged symptoms.^[4]^ Underlying medical conditions and risk factors were not associated with an extended duration of symptoms.

There are several limitations in this study. The experience in northern New Jersey and sample size may not be generalizable. Inherent to telephone surveys, data is subject to recall and nonresponse biases.

As children return to school, our study provides a better understanding of the clinical impact and populations at higher risk for COVID-19 to guide public health initiatives and help pediatricians tailor their anticipatory guidance. Larger studies addressing the clinical and psychosocial impact of CoVID-19 infection in children living in high-risk environments are warranted.

## Author contributions

Drs Denny and Shah equally contributed in the initial draft of the manuscript, conducted interviews, collected data, and reviewed and revised the manuscript.

Drs Petro, Choksey, Hintz, Rethi, Sanchez, Sylla and Ms DeSantis, conducted interviews, collected data, and reviewed and revised the manuscript.

Ms Chiu designed the REDCap database, collected data, carried out data analyses, and reviewed and revised the manuscript

Drs Gagliardo and Kairam, critically reviewed and revised the manuscript

Dr Eberechi Nwaobasi-Iwuh coordinated and supervised data collection and critically reviewed and revised the manuscript.

Dr Di Pentima conceptualized and designed the study, collected data, carried out data analysis, and critically reviewed the manuscript.

All authors approved the final manuscript as submitted and agree to be accountable for all aspects of the work.

**Conceptualization:** M. Cecilia Di Pentima.

**Data curation:** M. Cecilia Di Pentima.

**Formal analysis:** Stephanie Chiu, M. Cecilia Di Pentima.

**Investigation:** Vanessa Denny, Niva Shah, Karolina Karolina Petro, Karishma Choksey, Elizabeth DeSantis, Molly Hintz, Shruthi Rethi, Sarah Sanchez Sarah Sanchez, Bernadette Sylla, Stephanie Chiu, M. Cecilia Di Pentima.

**Project administration:** Stephanie Chiu.

**Resources:** Eberechi Nwaobasi-Iwuh.

**Supervision:** M. Cecilia Di Pentima.

**Writing – original draft:** Vanessa Denny, Niva Shah, Karolina Karolina Petro.

**Writing – review & editing:** Vanessa Denny, Niva Shah, Karolina Karolina Petro, Karishma Choksey, Elizabeth DeSantis, Molly Hintz, Shruthi Rethi, Sarah Sanchez Sarah Sanchez, Bernadette Sylla, Christina Gagliardo, Neeraja Kairam, Eberechi Nwaobasi-Iwuh, M. Cecilia Di Pentima.
